# Insulin signaling as a therapeutic mechanism of lithium in bipolar disorder

**DOI:** 10.1038/s41398-022-02122-6

**Published:** 2022-08-29

**Authors:** Iain H. Campbell, Harry Campbell, Daniel J. Smith

**Affiliations:** Centre for Clinical Brain Sciences, University of Edinburgh, Edinburgh, UK

**Keywords:** Bipolar disorder, Molecular neuroscience

## Abstract

In this paper, we propose that lithium may exert its therapeutic effect in bipolar disorder by acting on insulin signaling pathways. Specifically, we assess the importance of the phosphatidylinositol 3-kinase/Protein Kinase B (PI3K/Akt) insulin signaling pathway and we assess how the action of lithium on both glycogen synthase kinase-3 (GSK3) and the phosphatidylinositol cycle may lead to mood stabilization mediated by PI3K/Akt insulin signaling. We also highlight evidence that several other actions of lithium (including effects on Akt, Protein kinase C (PKC), and sodium myo-inositol transporters) are putative mediators of insulin signaling. This novel mode of action of lithium is consistent with an emerging consensus that energy dysregulation represents a core deficit in bipolar disorder. It may also provide context for the significant co-morbidity between bipolar disorder, type 2 diabetes, and other forms of metabolic illness characterized by impaired glucose metabolism. It is suggested that developments in assessing neuronal insulin signaling using extracellular vesicles would allow for this hypothesis to be tested in bipolar disorder patients.

## Introduction

Lithium is currently the most effective treatment for bipolar disorder (BD), but even after 60 years of research, its mechanism of action is not fully understood. Previously, we hypothesized that impaired glucose metabolism and mitochondrial dysfunction in BD, characterized by disrupted oxidative phosphorylation and increased glycolysis, are best explained as a result of disruption of a primary insulin signaling mechanism in the brain: the phosphatidylinositol 3-kinase/Protein Kinase B (PI3K/Akt) pathway [1]. Historically the brain has been considered to be insulin independent, however increasing evidence indicates that PI3K/Akt insulin signaling plays an important role in regulating glucose metabolism in the hippocampus, cerebellum, and olfactory bulb [2]. In this paper, we provide further support for this hypothesis by highlighting the effects of lithium on insulin signaling pathways which mediate glucose metabolism in the brain.

## Energy dysregulation and neuronal and glial cell insulin resistance

Energy dysregulation has been recognized as a core deficit in BD along several lines of evidence. At the macroscopic level, significant shifts in circadian rhythm, rest, and activity strongly indicate a dysregulation of energy generation and expenditure [3, 4]. At the microscopic level, growing evidence for mitochondrial dysfunction indicates disruption of energy production in the cell [5]. Concurrently, metabolic dysfunction characterized by insulin resistance (IR) and abnormal glucose metabolism are becoming increasingly recognized as important in the pathophysiology of bipolar disorder [6, 7]. Significant dietary and lifestyle changes over the past century have led to increased allostatic load on human physiology and a global epidemic of metabolic syndrome. The effects on peripheral tissues of the body are apparent with increasing prevalence of type 2 diabetes and related conditions. However, the effects of IR as a chronic stressor on the brain are only now beginning to be understood. For several decades the role of insulin in the brain was considered to be insignificant compared to its role in peripheral tissues. However, the presence of insulin receptors and insulin signaling mechanisms in the brain and their importance for neuronal function is now well established [8]. It has been demonstrated, both in vivo and in vitro, that insulin resistance occurs in neurons of the central nervous system (CNS) [9]. Insulin signaling plays a critical role in energy regulation in the body and brain; regulating the transport of glucose into cells and the production of cellular energy in the form of adenosine triphosphate (ATP). Neuron-specific insulin receptor knockout animal studies provide evidence for the importance of insulin signaling for neuronal function, energy metabolism, and mood-state. Insulin receptor knockout in the hippocampus of mice results in depression and anxiety-like behaviors, impaired cognition and metabolic abnormalities [10, 11].

Insulin signaling also plays an important role in regulating glucose metabolism in glial cells. Astrocytes express insulin receptors in the (IR)-B isoform [12]. Insulin resistance can be induced in astrocytes through the fructose induced insulin resistance cell model resulting in decreased insulin receptor and Akt phosphorylation [13]. Knockout of astrocyte insulin receptors in mice has been demonstrated to lead to decreased dopamine release and anxiety and depressive-like behaviors [14].

## Two leading hypotheses for BD pathophysiology: the GSK3 inhibition hypothesis and the inositol depletion hypothesis

**Fig. 1:**
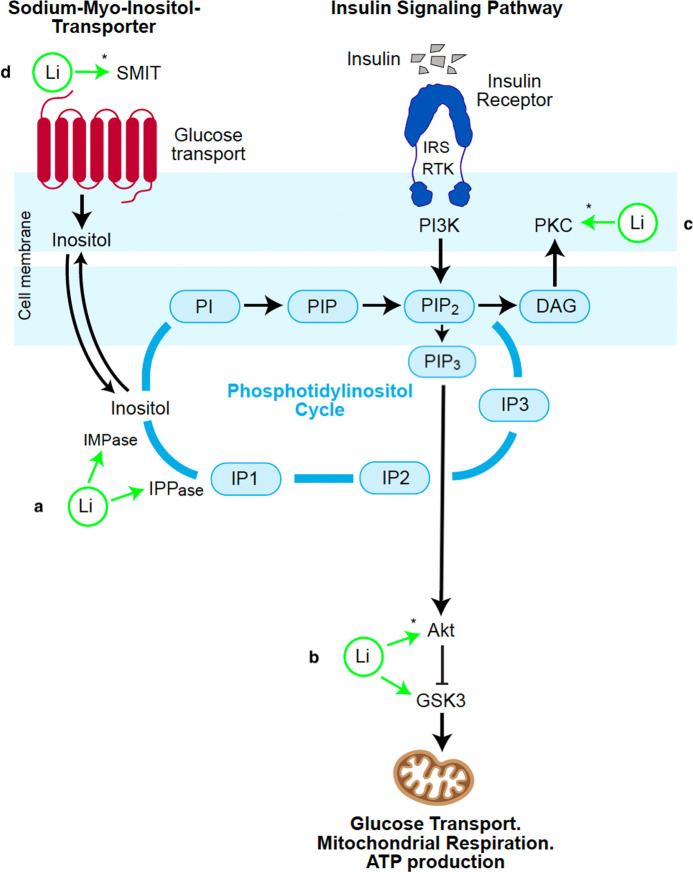
Direct and indirect targets of lithium which play a role in the PI3K/Akt insulin signaling pathway. **a** IMPase and IPP in the PI-Cycle. Lithium inhibits the activity of the PI-cycle in 2 areas: inositol monophosphatase (IMPase) and inositol polyphosphate-1-phosphatase (IIPase), thereby limiting the turnover rate of PIP2 available for PI3K signaling. **b** GSK3. Lithium inhibits GSK3 directly through uncompetitive inhibition of binding of the GSK3 cofactor magnesium and indirectly through activation of Akt. **c** PKC. Lithium acts to inhibit PKC translocation from the cytosol to the cell membrane. **d** SMIT. Lithium indirectly inhibits the activity and expression of SMIT, possibly through primary effects on the PI-Cycle. Li with an asterisk (*) indicates an indirect target of lithium. Li Lithium, IRS insulin receptor substrate, RTK tyrosine kinase adapter molecules, DAG diacylglycerol, PKC protein kinase C, SMIT sodium myo-inositol transporter, IMPase inositol monophosphatase, IPPase inositol polyphosphatase, IP1 inositol (1) phosphate, IP2 inositol bisphosphate, IP3 inositol (1,4,5) triphosphate, PI phophotidylinositol, PIP phosphatidylinositide phosphate, PI3K phosphatidylinositol 3-kinase, PIP2 phosphatidylinositol 4,5 bisphosphonate; PIP3 phosphatidylinositol 3,4,5 triphosphate, Akt protein Kinase B, GSK3 glycogen synthase kinase-3.

Reviews of the mechanism of action of lithium note a wide range of effects (such as upregulation of adenyl cyclase, cyclic adenosine monophosphate, brain-derived neurotrophic factor (BDNF), and bcl-2, and downregulation of sodium myo-inositol transporter, protein kinase C, and myristoylated alanine-rich C-kinase (MARCKS)) but the inhibitory effects of lithium on GSK3 and the phosphatidylinositol cycle (PI-cycle) have emerged as the leading hypotheses for therapeutic effects in BD [15]. These primary mechanisms have been investigated as independent phenomena, and discussion has focused on which may be more relevant to mood stabilization. However, GSK3 and the products of the PI-Cycle are both important components of PI3K/Akt insulin signaling and their function is tightly coupled through Akt (Fig. 1) [16]. Inositol metabolism is central to the function of the phosphatidylinositol 3-kinase pathway. Inositol phosphatases generated in the PI-Cycle interact with PI3K to generate second messengers which directly activate or inhibit the insulin signaling mechanism. The products of the PI-cycle diacylglycerol (DAG) and phosphatidylinositol 3,4,5 triphosphate (PIP3) are important mediators of insulin signaling and insulin resistance [17].

GSK3 plays a central role in this insulin signaling pathway and is directly influenced by Akt, thereby mediating many of the downstream mitochondrial and metabolic effects of insulin [18]. GSK3 is constitutively active and insulin signaling is achieved through transient phosphorylation and inactivation [19]. This temporary suppression of GSK3 in response to insulin allows for a diverse range of the metabolic effects of insulin to occur, such as glucose transport into the cell, conversion to pyruvate, and subsequent oxidative phosphorylation to produce energy (ATP) in the mitochondria [20].

## PI3K/Akt insulin signaling and insulin resistance

Activation of the PI3K/Akt pathway (Fig. 1) occurs when insulin binds to insulin receptors, leading to accumulation of insulin receptor substrates (IRS). The IRS then activate tyrosine kinase adapter molecules (RTK) which in turn activate PI3K. PI3K phosphorylates phosphatidylinositol 4,5 bisphosphonate (PIP2) to generate phosphatidylinositol 3,4,5 triphosphate (PIP3) which then activates Akt [21]. Akt phosphorylates Ser21 of GSK3α and Ser9 of GSK3β resulting in transient inactivation of GSK3β [22, 23]. The transient inactivation of GSK3β facilitates downstream metabolic and mitochondrial effects including upregulation of glucose transport, mitochondrial respiration, and ATP production [20, 24, 25]. GSK3 suppression therefore acts as a critical mediator of the effects of insulin on cell metabolism and mitochondrial function.

**Fig. 2:**
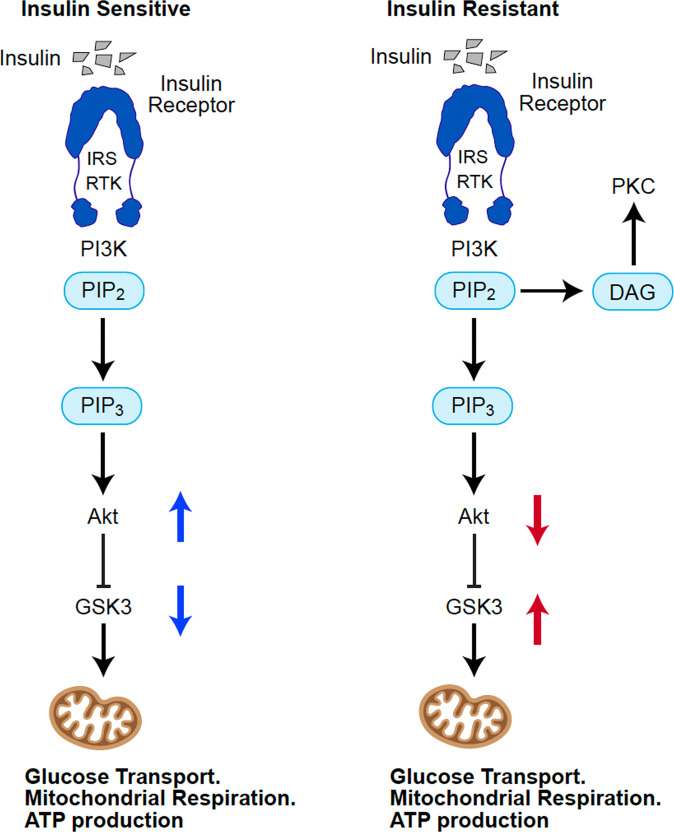
In the insulin-sensitive cell, activation of the PI3K/Akt insulin signaling pathway activates Akt which in turn phosphorylates GSK3 causing transient inhibition. Inhibition of GSK3 serves as a signal to activate downstream metabolic effects such as GLUT 4 mediated glucose uptake, glycogen synthesis, and mitochondrial respiration. In contrast, in the insulin-resistant cell PIP2 is cleaved to form DAG, activating PKC which phosphorylates and inhibits the insulin receptor. This inhibition of insulin signaling leaves GSK3 in a state of chronic overactivity with resultant deleterious effects on glucose metabolism and mitochondrial respiration. PI3K phosphatidylinositol 3-kinase, PIP2 phosphatidylinositol 4,5 bisphosphonate, PIP3 phosphatidylinositol 3,4,5 triphosphate, Akt protein Kinase B, GSK3 glycogen synthase kinase-3.

States of insulin resistance can occur through chronic overactivation and consequent blunting of this pathway. Such conditions can occur under prolonged hyperinsulinemia leaving the constitutively active GSK3 in a state of chronic overactivity as shown in Fig. 2 [26].

This state of metabolic dysfunction has been observed to occur in the CNS as well as in peripheral tissues [27]. Overactive GSK3 leaves the cell insensitive to insulin which regulates glucose metabolism through PI3K/Akt mediated suppression of GSK3. This disruption of normal metabolic function inhibits glucose metabolism and mitochondrial ATP production via reduced GLUT 4 mediated transport of glucose into the cell as well as inhibition of the pyruvate dehydrogenase complex and oxidative phosphorylation [24, 25]. Evidence of GSK3 hyperactivity and its association with mood state in BD is indicated by human and animal studies, however, the source of dysregulation has yet to be identified [28, 29]. Insulin resistance in the form of disruption of the PI3K/Akt pathway contextualizes several observations in the BD literature: high prevalence of insulin resistance, mitochondrial dysfunction, abnormal glucose metabolism, GSK3 overactivity, and the effect of lithium on the PI-cycle and GSK3.

## Metabolic effects of lithium

Lithium is not generally considered to exert therapeutic action through metabolic pathways. However, it has been demonstrated to have metabolic effects. Notably, it has been demonstrated to restore insulin sensitivity in animal models of insulin resistance, as summarized by Lee and Kim [30, 31]. Insulin-mediated glucose transport into the cell can be increased 2.5-fold in the presence of lithium [32]. And small molecule inhibitors of GSK3β have been demonstrated to lead to improvements in whole-body insulin sensitivity in insulin-resistant animals [33]. Impaired suppression of GSK3 is an established feature of insulin resistance both in peripheral tissues and in the CNS [2, 19]. There are several indications in the literature that the action of lithium in suppressing GSK3 and stabilizing mood may be mediated through insulin signaling. In addition to lithium directly inhibiting GSK3 through competitive binding with magnesium, it also acts through an independent effect on Akt, which is upstream of GSK3 in the PI3K/Akt insulin signaling pathway [34]. It has been demonstrated that Akt kinase activity is required for lithium to modulate mood-related behaviors in mice, suggesting that the therapeutic effect may occur via a larger signaling system as opposed to GSK3 alone [35]. Phosphorylation of insulin signaling proteins has been shown to increase in response to lithium treatment in an animal model of tricyclic antidepressant resistance, indicating increased insulin sensitivity [36]. In humans, insulin stimulation of peripheral blood mononuclear cells (PBMCs) resulted in phosphorylated GSK3 levels rising by 87% in those who respond strongly to lithium, compared to a 37% reduction in lithium non-responders [37]. The inability of insulin to suppress GSK3 as observed in the strong lithium responders may serve as an indication of a unique form of lithium-responsive metabolic dysfunction in bipolar disorder patients.

GSK3 is an important node at the intersection of multiple signaling pathways involved in metabolism, inflammation and cellular signaling. These include the Wnt pathway which regulates cell fate determination and migration [38] and toll-like receptor signaling pathways which regulate inflammation [39]. It is not possible therefore, to say conclusively that the effects of lithium on GSK3 necessarily imply an action on insulin signaling. However, the effects of lithium on the PI-Cycle and several other important insulin signaling mechanisms which will be discussed here provide further evidence that this pathway is an important mediator of GSK3 dysfunction in BD.

## PI3K and the phosphatidylinositol cycle

Inhibition of the PI-cycle is a well-studied hypothesis for the mechanism of action of lithium in BD. The PI-Cycle is a series of cyclical biochemical reactions that generate a variety of phosphoinositides from inositol maintained at a stable level. These phosphoinositides act as signaling molecules via interactions with a diverse range of external biological systems playing roles in cell survival, metabolism, autophagy, division and migration as well as vesicular trafficking and ion channel regulation [40–42].

Levels of inositol in the brain are significantly higher than in the blood and other tissues, suggesting an important role in neuronal function [43]. PI-cycle function is particularly important in the brain because inositol uptake at the blood-brain barrier is limited. De-novo synthesis of myo-inositol in the brain by the PI-Cycle provides almost all (97%) of the myo-inositol in the brain [44].

The PI-cycle plays a signficant role in PI3K/Akt insulin signaling generating the second messenger phosphoinositide PIP3 and diacylglycerol (DAG) which activate or inhibit insulin signaling respectively as Fig. 2 illustrates. The rate of turnover of PIP2 in the PI-Cycle appears to be the important factor determining the activation of the insulin signaling pathway [45].

Inhibition of the PI-cycle by lithium slows the turnover of PIP2 and reduces the pool available for generation of PIP3 and activation of the insulin signaling pathway. Disruption of these phosphoinositide second messengers have been observed in the occipital, temporal, and frontal cortex of bipolar subjects [46]. Membrane phospholipid levels of PIP2 have been observed to be elevated in manic BD patients compared to healthy controls [47]. In contrast, in euthymic patients PIP2 has been observed to be decreased relative to healthy controls [48]. Cycling from euthymic to manic state; the relative percentage of PIP2 in platelet membranes has been observed to increase in one case study, where it was possible to reduce the level of PIP2 again with lithium treatment [49]. Lithium inhibits the activity of the PI-cycle at two stages: inositol monophosphatase (IMPase) and inositol polyphosphate-1-phosphatase (IIPase), thereby limiting the turnover rate of PIP2 available for PI3K signaling (Fig.1). IMPase inhibitors such as Ebselen are a significant focus of research into new drug targets for BD [50]. In contrast, the antidepressant fluoxetine stimulates the PI-Cycle and increases the rate of PIP2 turnover [51].

PIP2 has two fates in the PI-cycle; it can go on to form PIP3 and activate insulin signaling or it can be cleaved to form DAG and inhibit the pathway (Fig. 2). The most extensive research on this mechanism has come from Peterson et. al who demonstrated in peripheral tissues that the blunting of insulin signaling in the presence of hyperinsulinemia occurs primarily through accumulation of DAG [12]. DAG inhibits insulin signaling via interaction with PKC, which is also indicated as a significant target of lithium.

The PI-cycle acts as a significant regulator of insulin signaling via its products DAG and PIP2. An overactive or underactive PI-cycle may be expected to create states of hyper or hypo-metabolism respectively via downstream effects on glucose metabolism and mitochondrial function.

## Protein kinase C (PKC)

Protein kinase C (PKC) has been associated with BD pathophysiology in many studies [52]. However, the effects of PKC on insulin signaling have not generally been considered relevant to BD. Peterson et. al show that DAG/PKC interaction is perhaps the most critical mediator of insulin resistance acting via the PI3K/Akt pathway [12]. Both lithium and valproate have been demonstrated to reduce PKC activity [53]. Levels of DAG are regulated by the activity of diacylglycerol kinase (DGK) which recycles DAG in the PI-Cycle. Reduced activity of DGK prevents the recycling of DAG thereby impairing the PI-cycle and insulin signaling. Several genome-wide association studies (GWAS) have identified variants in the gene coding for diacylglycerol kinase η (DGKH) as a genetic risk factor for BD [54]. DGK*β* knockout mice display impaired GSK3β signaling and hyperactive behavior similar to human mania and this is reduced by lithium treatment [55]. Increased expression of DGKH has been observed in the pre-frontal cortex of BD patients [56]. Since these post-mortem samples cannot reflect dynamic, changing mood states they may therefore be more likely to reflect gene expression associated with the predominant mood state of bipolar disorder, which is depression. If metabolic state is indeed linked to mood state then manic and depressed mood state may be reflected by contrasting genetic expression and metabolic markers as observed in these studies.

Impaired DGK leaves the PI-cycle activity free of its normal self-regulatory capacity and it may therefore alternate between states of overactivity through increased flux of phosphoinositides and under-activity due to depletion or decreased flux of phosphoinositides. Lithium acts primarily to stabilize overactivity of the PI-cycle which may explain its strong anti-manic effect [57, 58].

The effects of lithium on the PI-Cycle and PKC are well established, however, the mechanisms that may lead to mood stabilization are still unclear. Lithium-induced reduction of myo-inositol level is not always associated with mood stabilization and so the interactions of the second messenger phosphoinositides on downstream signaling mechanisms may have better explanatory scope [59]. Research examining the role of PIP3 and DAG as second messengers in BD has focused primarily on their effects on neurotransmission. We hypothesize that their metabolic role in insulin signaling may be an overlooked aspect of BD pathophysiology. A dysregulation of PIP2 and DAG production in the PI-Cycle would necessarily create states of energy dysregulation.

## Sodium myo-inositol transporters (SMIT) and the role of inositol

Sodium myo-inositol transporters (SMIT) are a proposed, indirect target of lithium. SMIT determines the level of available inositol in the cell to act as a substrate for insulin signaling and therefore plays an important role in metabolic function. A study on SMIT mRNA in BD reported a significantly higher expression of SMIT mRNA in untreated bipolar type 1 patients and that this was downregulated by treatment with lithium [60]. Upregulation of SMIT may reflect an increased demand for inositol due to hyperactive PI-cycle activity in bipolar type 1 patients who experience mania more frequently than those with type 2 BD. In the same study, expression of SMIT mRNA was not found to be increased in bipolar type 2 patients who experience depression more frequently. This may provide an indication that the manic state increases the demand for inositol to facilitate a hypermetabolic state. Lithium acting to inhibit hyperactivity of the PI-cycle would then necessarily reduce the demand for SMIT transport of inositol. Lithium uncompetitively inhibits IMPase and IPPase in the phosphatidylinositol cycle and so does not inhibit inositol metabolism unless it becomes active above a certain threshold [15]. Therefore, lithium may have little effect on states of depleted intracellular inositol but prove to be very effective in states of overactivity of the inositol cycle. This explanation is consistent with the stronger anti-manic than antidepressant effect of lithium. The uncompetitive inhibition lithium exerts has been noted in the literature to be an advantage over PI-cycle inhibitors which act indiscriminately to supress PI-cycle activity [51].

Glucose and inositol are transported into the cell by SMIT and hyperglycemia has been shown to inhibit SMIT uptake of inositol [61]. Additionally, hyperglycemia inhibits re-uptake of myo-inositol in the kidney and increases urinary excretion of myo-inositol. Excretion of myo-inositol is common in IR conditions and urinary detection of myo-inositol is used as a diagnostic tool [62]. Metabolomic evidence of altered inositol phosphate metabolism in urine samples from BD patients has been observed, although more data is needed to establish this [63]. In addition to preventing inositol from reaching the CNS, hyperglycemia and insulin resistance alter the ratio of inositol isomers in tissues. Detection of changes in these isomer ratios in blood and urine are used as early markers of insulin resistance in several metabolic conditions [64]. In conditions of insulin resistance such as polycystic ovary syndrome (with which BD is significantly comorbid) inositol supplementation is a standard treatment and has been demonstrated in several randomized controlled clinical trials to improve insulin sensitivity and lower blood glucose [65]. The leading hypothesis for this effect of inositol is that it acts to restore inositol substrate for PI3K/Akt insulin signaling [61]. A small trial of inositol for bipolar depression reported 50% or greater decreases in MADRS scores in 8 of the 12 patients taking inositol [66]. And reduced brain myo-inositol is also associated with major depressive disorder (MDD) [67], however, trials of inositol supplementation for MDD are inconclusive [68].

Acute experimental diabetes reduces the concentration of free myo-inositol in peripheral nerves in both animals and humans [69, 70]. Hyperglycemia and hyperinsulinemia-mediated inhibition of inositol uptake to the CNS may leave the CNS deprived of inositol thereby inhibiting insulin signaling. Barkai et al. report that CNS inositol levels were depleted in samples from hospitalized BD patients [71]. It may be expected that under such conditions the brain upregulates de-novo synthesis of inositol to compensate. A spectrophotometric study showed that G6PD which facilitates the de-novo synthesis of myo-inositol was positively correlated with a marker of mitochondrial dysfunction in BD. This result may indicate that G6PD-mediated inositol synthesis plays a role in a compensatory mechanism for mitochondrial dysfunction in BD [72]. There is limited research on SMIT and its interaction with lithium in BD and so only modest conclusions can be drawn at this time. However, the significant role of SMIT in facilitating PI-cycle activity and its relevance to insulin resistance provide further support for a metabolic component to lithium action.

## PI3K insulin signaling tests and CNS biomarkers

Markers of insulin signaling can be measured in peripheral blood mononuclear cells as performed by Tye et al. and such approaches may have the potential to lead to a treatment response biomarker [37]. Extracellular vesicles of neuronal origin have also emerged as a viable method to assess neuronal insulin signaling. The PI3K/Akt/GSK3β pathway has been studied using this method in Alzheimer's Disease and has been shown to contribute to neurodegeneration [2]. Several tests and biomarkers have been developed to establish evidence of neuronal insulin resistance in these studies, some of which may be useful for BD research. Peripheral insulin resistance can be measured by HOMA-IR, however, it has been noted that this may not be entirely suitable for CNS assessment. Blood-brain barrier transport of insulin may be impaired and therefore peripheral IR therefore may not reflect neuronal IR [73]. Indeed, CNS IR has been demonstrated to occur independently and in advance of peripheral IR [74]. For this reason, intranasal insulin paired with MRI, EEG, and MEG brain imaging is used to examine the effects of insulin on the brain directly [75]. Intranasal insulin bypasses the blood-brain barrier and allows for direct uptake of insulin to the brain. Failure of insulin to induce a response in neuroimaging is considered a sign of insulin resistance [76]. There are currently no standardized measurements for this method however, making it difficult to interpret results and compare them across studies. Intranasal insulin has not been widely used in BD research, however, a clinical trial did show that it was associated with an improvement in neurocognitive function in euthymic patients with BD [77].

The primary measurement obtained in intranasal insulin experiments is the phosphorylation of insulin receptor substrates [75]. The ratio of serine-phosphorylated insulin receptor substrate to total phosphorylated insulin receptor substrate indicates the activation of the insulin signaling pathway. Additionally, the phosphorylation of the downstream targets PI3K, Akt, and GSK3β can be measured.

The need for MRI and delivery of insulin makes this test difficult to perform, however, new alternatives are emerging. Kellar’s 2020 review highlights measuring the same phosphorylation in IRS derived from neuronal-enriched extracellular vesicles in plasma as a promising and non-invasive approach for future research [78]. Extracellular vesicles of neuronal origin are particles released by neurons that cross the blood-brain barrier and can be detected in peripheral blood. Techniques, which study these vesicles by means of immunoprecipitation methods, have been used to assess neuronal insulin signaling extensively in Alzheimer’s disease and to a lesser extent in schizophrenia and depression. The first study using this technique in BD research was reported in 2021 by Mansur et al. [79]. They assessed neuronal insulin signaling at its first node (IRS-1) and along the canonical pathway (Akt, GSK3β, p70S6K). An association was found between IRS-1 phosphorylation and increased cognitive dysfunction as well as reduced hippocampal volume. Due to the minimal invasiveness and relative ease of the sample collection; testing of extracellular vesicles could prove a useful tool for assessing the risk of onset of bipolar disorder in those who are susceptible. Additionally, testing of extracellular vesicles could have diagnostic, treatment stratification, and disease-modifying implications. Impaired neuronal insulin signaling may represent a specific form of bipolar disorder amenable to treatment with insulin-sensitizing medications, and such tests could identify those who are likely to respond.

## Insulin resistance and response to lithium

Calkin et. al noted a strong association between IR and BD as well as a strong antidepressant effect of insulin-sensitizing medication Metformin in BD [6, 80]. However, they also noted that patients with more significant IR were less likely to respond well to lithium. Across the euglycemic, insulin resistance, and type 2 diabetic groups partial response rates to lithium were similar at around 40% in each group. However, the rate of full response to lithium was higher in the euglycaemic group (54.8%) and much lower in the type 2 diabetes group (21.1%). This may indicate that a full response becomes less likely in more severe cases of IR. Neuronal IR can occur independently and in advance of peripheral IR (HOMA-IR) and so further work to determine neuronal IR and response to lithium may provide further valuable insights. In contrast, a mechanistic study of dynamic insulin stimulation of peripheral blood mononuclear cells in BD patients showed that lithium-responsive patients could be identified by markers of disrupted insulin signaling [37]. These contrasting results point to a relationship between IR and lithium response. However, more research is required to understand the dynamics of these observations and investigate reliable markers before any implications for treatment stratification can be considered. Given the demonstrated effects of selective serotonin re-uptake inhibitor fluoxetine on metabolic mechanisms [51] this line of research may also have the potential to identify candidates for lithium augmentation during antidepressant treatment. An insulin-like effect of lithium was noted in one of the first papers to demonstrate that lithium acts on GSK3 in bipolar disorder. The paper by Klein et.al stated “Lithium mimics insulin action…and while the mechanism remains unknown, we suggest that lithium mimics insulin by inhibiting GSK3…” [81]. If lithium exerts an insulin-like activity then this would be expected to facilitate downstream metabolic effects on glucose transport, mitochondrial respiration, and ATP production. Animal studies [30, 31] indicate that the action of lithium is best described as an insulin sensitizer, increasing sensitivity of the cell to insulin, with insulin itself remaining the primary activator of the PI3K/Akt pathway. Certainly, in more severe cases of insulin resistance reduced phosphorylation of the insulin signaling pathway components are observed and so increased levels of insulin are required for activation and downstream metabolic effects [75]. This may also be true of lithium if it acts as an insulin sensitizer, indicating that weakened response to lithium would be expected in more severe cases of IR and that higher dosage may be required to achieve pathway activation.

## Conclusions

Disruption of the PI3K/Akt insulin signaling pathway has wide explanatory scope for several features of BD. It unites several important mechanisms of lithium action within a coherent context; the PI-cycle and GSK3β are linked via this pathway, as well as effects on SMIT and PKC. Insulin resistance is defined as a blunting of the PI3K insulin signaling pathway and chronic overactivation of GSK3β is an expected outcome of this state. Chronic GSK3β overactivation of this kind has been shown to disrupt brain energy regulation via impairment of glucose metabolism and mitochondrial function. While regulation of brain glucose metabolism has historically been regarded as largely insulin-independent, increasing evidence indicates that PI3K/Akt insulin signaling plays an important role in the hippocampus and cerebellum, and these brain regions have been identified as particularly vulnerable sites of gray matter loss in BD [82].

The significant acceleration in the increase in burden of disease from BD in recent decades may require an explanation which extends beyond genetic causes and takes into account increasing allostatic load from environmental stressors [83]. Significant shifts in behavior related to diet and physical activity over the past 100 years have led to a global epidemic of insulin resistance-related conditions [84]. The effects on peripheral tissues of the body are apparent with increasing prevalence of metabolic syndrome and type 2 diabetes. However, the effects of insulin resistance as a chronic stressor on the brain and central nervous system are only beginning to be understood.

The insulin-sensitizing effects of lithium via its action on several nodes of the insulin signaling pathway (GSK3, PI-Cycle, Akt) have been demonstrated but are not generally considered relevant to mood stabilization in BD. However, when BD is conceptualized as a disorder of energy regulation, these mechanisms become highly plausible because of their effects on mitochondrial function, circadian rhythm, and glucose metabolism.

We hypothesize that in a proportion of BD patients insulin resistance acts as a chronic stressor leading to impairment of PI3K/Akt insulin signaling in the brain. The resultant chronic overactivation of GSK3 and dysregulation of glucose metabolism in neurons leads to mitochondrial dysfunction and energy dysregulation, manifesting as manic or depressive symptoms. Lithium acts on several stages of the PI3K/Akt insulin signaling pathway to restore sensitivity to insulin, resulting in improvements in downstream glucose metabolism and mitochondrial function. The targets of lithium action discussed in this paper lie at the intersection of many signaling pathways and mechanisms which regulate metabolism, inflammation and immune response. There may therefore be a wide variety of immuno-metabolic stressors which play a role in BD onset in those who are genetically susceptible. Increased IR appears to be the most significant environmental shift which could explain increasing burden of disease of BD worldwide [84]. This hypothesis merits further study since these pathways may be amenable to dietary and/or other therapeutic interventions as an adjunct to current treatments in BD.

It is now possible to assess neuronal insulin signaling using extracellular vesicles and a study in bipolar patients has established preliminary evidence for impaired PI3K/Akt insulin signaling [79]. We suggest that future studies consider measuring biomarkers related to mitochondrial function and glucose metabolism (lactate, pyruvate, and citric acid cycle metabolites) as well as markers of insulin signaling (IRS, PI3K, Akt, GSK3β, mammalian target of rapamycin (mTOR)) in neuronal-enriched extracellular vesicles to gain insight into the effects of lithium on insulin signaling. A recent randomized, quadruple-masked, placebo-controlled clinical trial by Calkin et.al noted significant improvement of bipolar depression in patients given insulin sensitizer metformin over 26 weeks [80]. Based on the high prevalence of IR in BD and the effects of lithium on key components of the PI3K/Akt insulin signaling pathway, insulin-sensitizing agents should be considered for further study in BD, both for their metabolic health effects and for their potential to stabilize mood.
